# Modelling wound area in studies of wound healing interventions

**DOI:** 10.1186/s12874-024-02326-y

**Published:** 2024-09-16

**Authors:** Samuel I. Watson, Eleni Gkini, Jon Bishop, Katie Scandrett, Indra Napit, Richard J. Lilford

**Affiliations:** 1 Institute of Applied Health Research, University of Birmingham, Birmingham, B15 2TT UK; 2 The Leprosy Mission Nepal, Kathmandu, Nepal

**Keywords:** Statistical modelling, Randomised controlled trial, Wounds, Ulcer

## Abstract

**Background:**

Experimental studies of wound healing often use survival analysis and time to event outcomes or differences in wound area at a specific time point. However, these methods do not use a potentially large number of observations made over the course of a trial and may be inefficient. A model-based approach can leverage all trial data, but there is little guidance on appropriate models and functional forms to describe wound healing.

**Methods:**

We derive a general statistical model and review a wide range of plausible mathematical models to describe wound healing. We identify a range of possible derived estimands and their derivation from the models. Using data from a trial of an intervention to promote ulcer healing in patients affected by leprosy that included three measurement methods repeated across the course of the study, we compare the goodness-of-fit of the models using a range of methods and estimate treatment effects and healing rate functions with the best-fitting models.

**Results:**

Overall, we included 5,581 ulcer measurements of 1,578 unique images from 130 patients. We examined the performance of a range of models. The square root, log square root, and log quadratic models were the best fitting models across all outcome measurement methods. The estimated treatment effects magnitude and sign varied by time post-randomisation, model type, and outcome type, but across all models there was little evidence of effectiveness. The estimated effects were significantly more precise than non-parametric alternatives. For example, estimated differences from the three outcome measurements at 42-days post-randomisation were − 0.01 cm^2^ (-0.77, 0.74), -0.44 cm^2^ (-1.64, 0.76), and 0.11 cm^2^ (-0.87, 1.08) using a non-parametric method versus − 0.03 cm^2^ (-0.14, 0.06), 0.06 cm^2^ (-0.05, 0.17), and 0.03 cm^2^ (-0.07, 0.17) using a square-root model.

**Conclusions:**

Model-based analyses can dramatically improve the precision of estimates but care must be taken to carefully compare and select the best fitting models. The (log) square-root model is strongly recommended reflecting advice from a century ago.

**Supplementary Information:**

The online version contains supplementary material available at 10.1186/s12874-024-02326-y.

## Introduction

Ulcers, traumatic wounds, burns and other open wounds present serious health risks to patients. The wound can become infected and without respite and treatment, wounds and ulcers can grow and cause deformities and issues with mobility. Social stigma is also associated with ulcers and related wounds. Medical interventions that can improve healing times and rates are therefore highly desirable.

As with many other types of medical intervention, experimental methods, particularly randomised controlled trials (RCT), present the best way of establishing what works to promote wound healing. There have been a large number of randomised trials and other studies of interventions to promote wound healing: for examples of systematic reviews in this area see [1–5]. Time to healing outcomes are frequently used in evaluations of wound healing interventions to estimate hazard ratios. However, there are many other estimands that may be relevant, such as differences in wound area or the proportion of wounds healed at a given time post study entry. Some trials use relatively complex derived outcomes, although often without clear justification. For example, previous studies have used: mean difference in proportionate reduction in wound area or a derived effect such as the mean difference in the proportion of patients who achieve a pre-specified proportionate reduction in wound area, mean difference in absolute wound area reduction or a relative ratio, the odds ratio or relative risk comparing the proportion of patients whose wounds had healed by a pre-specified time, and time to healing, among others. Almost exclusively, previous studies using outcomes based on wound area, rather than time-to-healing, use non-parametric estimators (like a difference in means or proportions) that compare mean values at a fixed time point. While these estimators have relatively weak assumptions, one limitation in this context is that they do not incorporate measurements made between baseline and the final study endpoint, and so do not make use of all the available data.

A *model-based* analysis may be of interest in studies of interventions to promote wound healing as it can “combine” all the observations made during the study. However, it must account for the healing rate of the wound likely not being linear with respect to time, measurement error, and repeated measures on the same wounds. There are multiple treatment effects that can be derived from these models. Yet there is little guidance available on best practice for wound area modelling.

There are several plausible mathematical models that describe the (proportionate) area of a healing wound as a function of time. The earliest reference we identified on the topic was published in 1916, which suggested a model including a linear function and square root of time [6]. There have been very few similar comparisons in the time since. Cukjati et al. compared a range of models for wound healing using data from a study of electrical stimulation of chronic wounds to promote healing [7]. They included exponential, Gompertz, and logistic type functions. Their models included an additional delay between the development of the wound and the start of the healing process. Their methods of model comparison were relatively limited and based on statistical significance of tests of residual sums of squares. This approach may be limited compared to the Bayesian methods we use in this article [8]. They concluded an exponential decay model was the best fitting. Wallenstein et al. fit Gompertz-like functions to data describing the healing of pressure ulcers in an RCT, although they did not compare the goodness-of-fit to other possible specifications [9]. Finally, Gorin et al. fit a linear, additive model to data following the healing of venous stasis ulcers to estimate the association between different aspects of the wound and its healing rate, although again they did not examine the goodness-of-fit of their models [10]. 

The aim of this article is to describe and compare models of wound healing and estimators of the effects of interventions to promote wound healing. We identify the range of relevant estimands and describe their estimation from models that allow for measurement error and cohort effects. We re-analyse data from a RCT of an intervention to promote healing of neuropathic ulcers in patients affected by leprosy. We compare the goodness-of-fit using a variety of methods and compare treatment effect estimates from the best-fitting models to illustrate the use of the proposed methods.

## Methods

### Wound area statistical models

Our objective is to model the area of a wound over the course of a study with the intention of estimating the effects of an intervention designed to improve healing times and rates. We make some assumptions:

(i) The wound begins (or continues) healing once the patient has entered the study (i.e. there is no lag phase);
(ii) The area of the wound is measured with random error proportionate to the area of the wound. Wound area is typically ascertained by imaging the wound and then using software to identify and trace its borders. So, both the cumulative size of deviations when tracing a wound’s boundary and the number of opportunities for such deviations will be greater for larger wounds.
(iii) Random variation in ulcer healing is proportionate to wound area.
(iv) The wounds heal, on average, following a common function describing the relationship between *proportionate* wound area and time from entry to the study.

If $\:{y}_{i}\left(t\right)$ represents the observed area of patient $\:i$’s wound at time $t\,{\in}\,[0,T)$ then the above assumptions imply the following data generating process:

$$\:{y}_{i}\left(t\right)={y}_{i}^{*}\left(0\right)f\left(t;\rho\:\right){u}_{i}\left(t\right)$$

where $\:{y}_{i}^{*}\left(0\right)$ represents the true (i.e. without measurement error), but unknown, wound area at the time of first measurement $\:t=0$, $\:{u}_{i}\left(t\right)$ represents the random error including measurement error, and $\:\rho\:$ represents the parameter(s) of the healing rate function. This model is linear on the log scale and so leads us to a statistical model:

$$\:\text{log}{y}_{i}\left(t\right)={w}_{i}\left(0\right)+\text{log}f\left(t;\rho\:\right)+{\epsilon}_{i}\left(t\right)$$

where $w_{i}(t)=\mathrm{log}(y^{*}_{i}(t))$ and $\epsilon_{i}(t)=\mathrm{log}(u_{i}(t))$ . We can assign distributions to the unknown components: $w_{i}(0){\sim}N({\mu}\,{\tau}^{2})$ and $\epsilon_{i}(t){\sim}N(-\frac{w^{2}}{2},w^{2})$ where the mean of this distribution is used to ensure $E(u_{i}(t))=1$, i.e. that the measurements are correct on average. We can gather terms for our final, parameterised model:

$$\:\text{log}{y}_{i}\left(t\right)={\beta\:}_{0}+{\alpha\:}_{i}+\text{log}f\left(t;\rho\:\right)+{e}_{it}$$

Where $\:{\alpha\:}_{i}\sim N\left(0,{\tau\:}^{2}\right)$ is an individual random effect and $\:{e}_{it}\sim N\left(0,{\omega\:}^{2}\right)$ is the random error term.

Table 1 shows the different specifications of $\:f\left(t;\rho\:\right)$ we compare. Mathematical models of wound healing and similar physiological phenomena that consider the underlying behaviour of relevant processes like hormone release by platelets and macrophages, and angiogenesis, suggest a non-linear function that heals fast and slows down as the wound decreases in size. For example, models of tumour growth often include exponential or Gompertz functions [11, 12]. An autoregressive model would also be relevant on this basis. Empirical analyses in the early 20th Century suggested that functions of time and its square root well describe wound area with respect to time [6, 13]. A simple model of a circle in which the radius decreased linearly with time would imply a quadratic function of time. In all these cases, one may argue that the function applies on the absolute or relative area scale, and so we include both linear and log versions.

**Table 1:** Healing rate functions describing the relationship between time and proportionate wound area. Time $\:t$ is assumed to be positive

Model	Function	Specification$\:\varvec{f}\left(\varvec{t};\varvec{\rho\:}\right)$
1	Exponential	$\exp(-\rho t)$
2	Gompertz	$\exp(1-exp\mathit\;(pt))$
3	Linear	$1\;+\;pt$
4	Quadratic	$1+p_1\;t+p_2\;t^2$
5	Square root	$1+p_{1\;}t+p_2\sqrt t$
6	Wendland 0	$\left(1-t\right)^p$
7	Wendland 1	$\left(1+\left(p+1\right)t\right)\left(1+t\right)^{p+1}$
8	Semiparametric^a^	$1-\sum\limits_m^{}p_mh\left(t\right)$
9	Log-linear	$\exp\left(1+pt\right)$
10	Log-quadratic	$\exp\left(1+p_1t+p_2t^2\right)$
11	Log-square root	$\exp\left(1+p_1t+p_2\sqrt{\mathit t}\right)$

^a^In this specification h(t) represent basis functions

We also include two “compactly supported” functions: “Wendland 0” and “Wendland 1” after their proposer [14]. These are functions that feature an exponential-type decay, but where a value of zero can be achieved. The two functions we include have this property but also only on a compact support $t\,{\in}\,[0,1]$: they reach a value of zero when $\:t=1$ [15]. We must therefore transform our time variable to the range [0,1] by dividing by a maximum value $\:{t}_{MAX}$ beyond which we expect all ulcers to have healed. Finally, a semiparametric approach may be preferred given that the true functional form is unknown. For the applied analyses below we select degree-3 basis functions for the splines and select five knot values spaced evenly over the time range.

To incorporate the effect of an intervention versus control, the healing rate function parameters vary by treatment and control status. To make this explicit, we notate the function $\:{f}_{1}\left(t;{\rho\:}_{1}\right)$ for treatment and $\:{f}_{0}\left(t;{\rho\:}_{0}\right)$ for control.

### Treatment effects

One of the advantages of using model-based approaches in interventional studies is that we can use all measures of wound area over the course of the trial to estimate a treatment effect, as opposed to comparing average area at a pre-specified time point. An obvious output from the analysis would be a graphical comparison of estimated healing rate functions. Other estimands that compare the outcomes in intervention and control conditions may include the following population-level summary measures, where $\:d=1$ indicates treatment allocation and $\:d=0$ control.

*Mean difference in wound area at time T*

$$\:{\delta\:}_{1}=E\left[{y}_{i}\left(t\right)|d=1\right]-E\left[{y}_{i}\left(t\right)|d=0\right]=E\left[{y}_{i}^{*}\left(t\right)\right]\left({f}_{1}\left(t\right)-{f}_{0}\left(t\right)\right)=\text{exp}\left({\beta\:}_{0}+\frac{{\tau\:}^{2}+{\omega\:}^{2}}{2}\right)\left({f}_{1}\left(t\right)-{f}_{0}\left(t\right)\right)$$

*Mean difference in proportionate wound area at time*

$$\:{\delta\:}_{2}=E\left[\frac{{y}_{i}\left(t\right)}{{y}_{i}^{*}\left(0\right)}|d=1\right]-E\left[\frac{{y}_{i}\left(t\right)}{{y}_{i}^{*}\left(0\right)}|d=0\right]={f}_{1}\left(t\right)-{f}_{0}\left(t\right)$$

#### Other treatment effects

In the Supplementary Information we derive similar expressions for the differences in healing *rates* up to time *T*. We can also estimate a survival function and related effects, including differences in the proportion of healed wounds up to time $\:T$, which is also shown in the Supplementary Information.

### Model fitting and comparison

We fit all the statistical models described. We use a Bayesian approach to model estimation and comparison. We fit the models using Markov Chain Monte Carlo (MCMC) using the Stan probabilistic programming language [16]. As described below, there were three separate measures for each wound in the data used in this article. We fit them all separately to compare model fit.

We use the leave-one-out cross validation score (LOO-CV) and widely applicable information criterion (WAIC) to compare the models for goodness of fit [17]. These criteria can only provide comparisons of models using the same data, and not between models fit with different data (i.e. outcomes from different measurement software). To compare models more broadly we also conduct a series of graphical posterior predictive model checks [8]. For each model we sample from the posterior predictive distributions of the wound area over time and graphically compare with the density of the data.

### Prior distributions

To complete the model specification we require the priors and hyperpriors. We use weakly informative priors, which constrain the parameters to a plausible range but provide little information within this range. We specify $\:\mu\:\sim N\left({\text{1,2}}^{2}\right)$, $\:\tau\:\sim N\left({\text{0,2}}^{2}\right)\left[0,\infty\:\right)$, and $\:\omega\:\sim N\left({\text{0,0.5}}^{2}\right)\left[0,\infty\:\right)$. These priors constrain the mean baseline ulcer area $\:\mu\:$ is between approximately 0 and 20 cm^2^ determined by the inclusion criteria (see Data below), and the variance parameters’ hyperpriors allow for relatively large values including noise of +/- 100%.

## Data

We re-analyse data from an individual-level randomised controlled trial of leukocyte and platelet rich fibrin gel (LPRF; treatment) versus standard saline dressing (control) to promote ulcer healing in patients affected by leprosy. Patients were randomly assigned to receive either treatment or control in a 1:1 ratio. The ulcer dressings were changed every three or four days (twice weekly) during the trial at which point the ulcer was imaged for its area to be measured. The objectives of our re-analysis of these data are: (i) compare the range of models to identify the best fitting model(s); and (ii) estimate and critically appraise the range of treatment effect estimates derived from the best fitting model(s).

Full details of the trial methods are published elsewhere, [18] we provide a brief summary here. Overall, 130 patients were enrolled in the trial. Informed consent was obtained from all participants. The inclusion criteria included that the ulcer area at enrolment was between 2cm^2^ and 20cm^2^. Two primary outcomes were specified: ulcer area at 42 days post-randomisation and time to complete healing. For the former outcome, the area of each ulcer was measured in three ways by two independent assessors. First, an image of the wound was taken using a Tablet computer device following a standardised protocol, which incorporated a measuring rule in the image. The area was then estimated by an independent assessor using “PUSH” software. For the other two methods, an image of the wound was taken using an “ARANZ” device, which is designed to capture high-resolution images of wounds and includes laser-based guides for establishing its position in 3D space. The area of the wound can then be measured in one of two ways: using the ARANZ software and manually tracing the wound or having the ARANZ software automatically detect the wound boundary and estimate the area. We refer to these methods as “ARANZ Manual” and “ARANZ Automatic”, respectively. Healing (complete re-epithelialisation of the ulcer) was assessed by the treating clinician and the independent assessors. Patients remained in the trial up to 70 days post-randomisation after which healing times would be right-censored and no more measurements taken. The maximum duration of the trial was 72 days. We choose $\:{t}_{MAX}=100$ for the analyses here: the function has value zero at $\:{t}_{MAX}$ and not all ulcers may be healed by trial end, so setting $\:{t}_{MAX}$ to larger than the total trial time allows for non-zero values.

## Results

Overall, our dataset contains 5,581 ulcer measurements of 1,578 unique images from 130 patients in the trial between zero- and 70-days post-randomisation. The mean baseline ulcer sizes from the three different measurement methods were 4.57 cm^2^ (ARANZ Manual), 4.20 cm^2^ (ARANZ Automatic), and 4.07 cm^2^ (PUSH). Summary statistics for the trial data are published in the main trial report.[submitted].

**Fig. 1:**
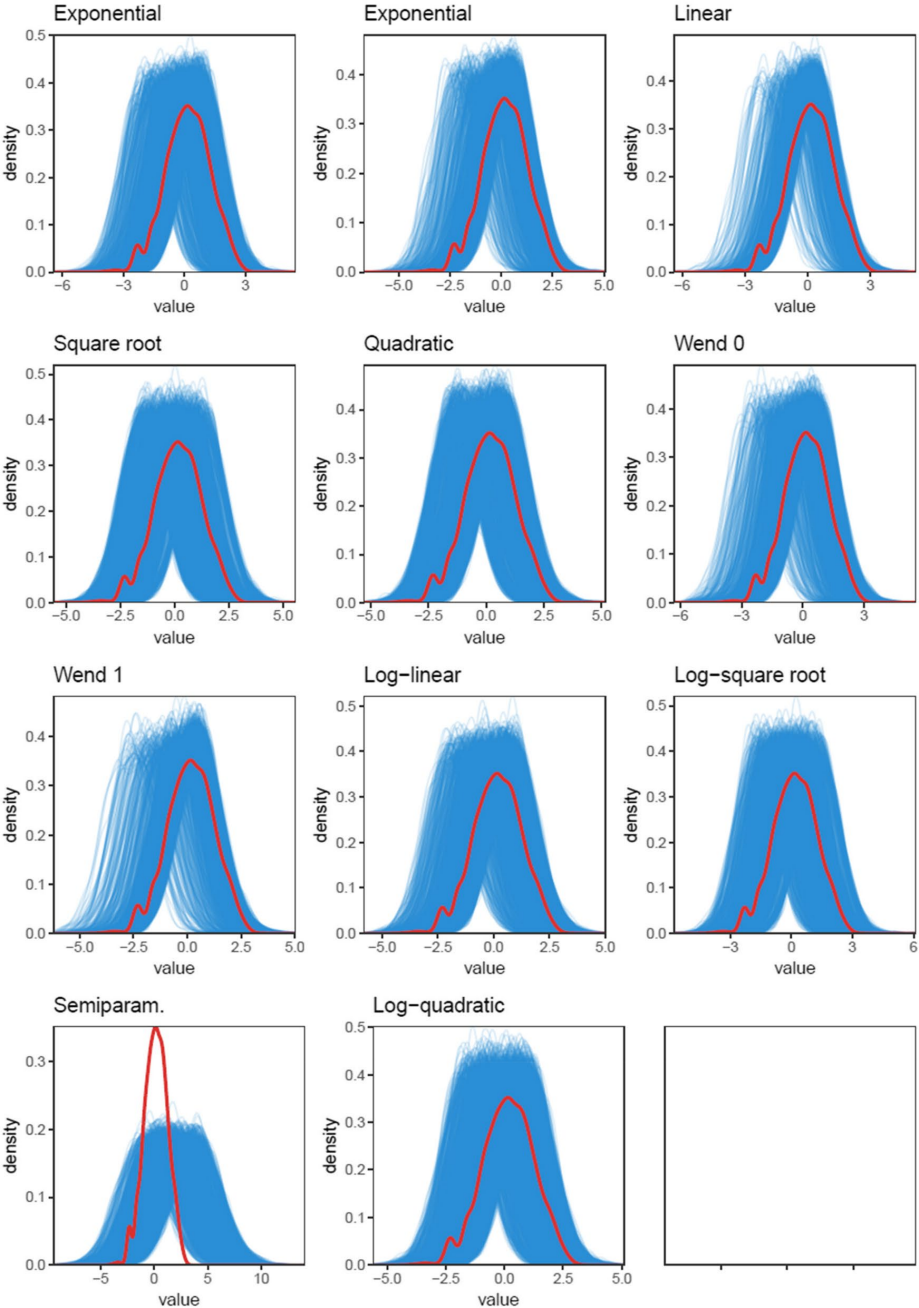
Graphical posterior predictive model checks for the ARANZ Automatic data. Blue lines represent samples from the posterior predictive distribution and the red line shows the density of the data

Table 2 reports the goodness of fit statistics for the different models and Fig. 1 shows the posterior predictive model checks for ARANZ Automatic data, the other posterior predictive model checks are shown in the Supplementary Information. The relative ordering of models in terms of goodness-of-fit was approximately the same across all three outcomes. The best three fitting models in all cases were the log-square root, log-quadratic, and square root functions, although the single best performing model differed between outcomes. The Gompertz function and compactly supported functions (Wendland 0 and 1), performed the worst. Graphically, the models revealed relatively large predictive uncertainty with the log-square root and log-quadratic models appearing to provide the most parsimonious set of predictive data sets. The semiparametric model overestimated the variance in the data relative to the other models.

**Table 2:** Goodness of fit statistics for the different models continued. The three best fitting models are in bold

Model	Outcome measure	LOO-CV	WAIC
Exponential	ARANZ Manual	50,861.04	39,532.72
Gompertz		33,343.06	24,782.54
Linear		18,859.66	14,619.62
Quadratic		46,253.63	35,023.48
Square root		**56**,**276.46**	**44**,**050.30**
Wendland 0		38,931.13	29,467.40
Wendland 1		27,546.86	20,496.48
Semiparametric		45,445.80	38,364.32
Log-linear		51,670.29	39,954.78
Log-square root		**59**,**979.63**	**43**,**483.61**
Log-quadratic		**56**,**170.34**	**43**,**947.12**
Exponential	ARANZ Automatic	48,035.34	37,359.80
Gompertz		32,060.78	23,299.23
Linear		18,602.89	14,043.35
Quadratic		44,084.41	32,863.08
Square root		**53**,**363.95**	**42**,**715.13**
Wendland 0		36,639.66	27,092.26
Wendland 1		24,862.11	18,366.37
Semiparametric		40,965.34	34,337.78
Log-linear		48,016.31	37,298.21
Log-square root		**52**,**868.86**	**41**,**384.16**
Log-quadratic		**52**,**703.47**	**42**,**295.78**
Exponential	PUSH	37,709.15	24,471.70
Gompertz		24,275.96	18,229.65
Linear		14,111.45	11,592.73
Quadratic		32,488.15	23,452.82
Square root		**40**,**212.90**	**29**,**386.88**
Wendland 0		28,516.35	21,235.42
Wendland 1		20,404.63	15,812.56
Semiparametric		37,784.01	30,014.45
Log-linear		37,224.10	27,337.31
Log-square root		**39**,**154.33**	**29**,**054.99**
Log-quadratic		**38**,**912.37**	**29**,**058.61**

**Fig. 2:**
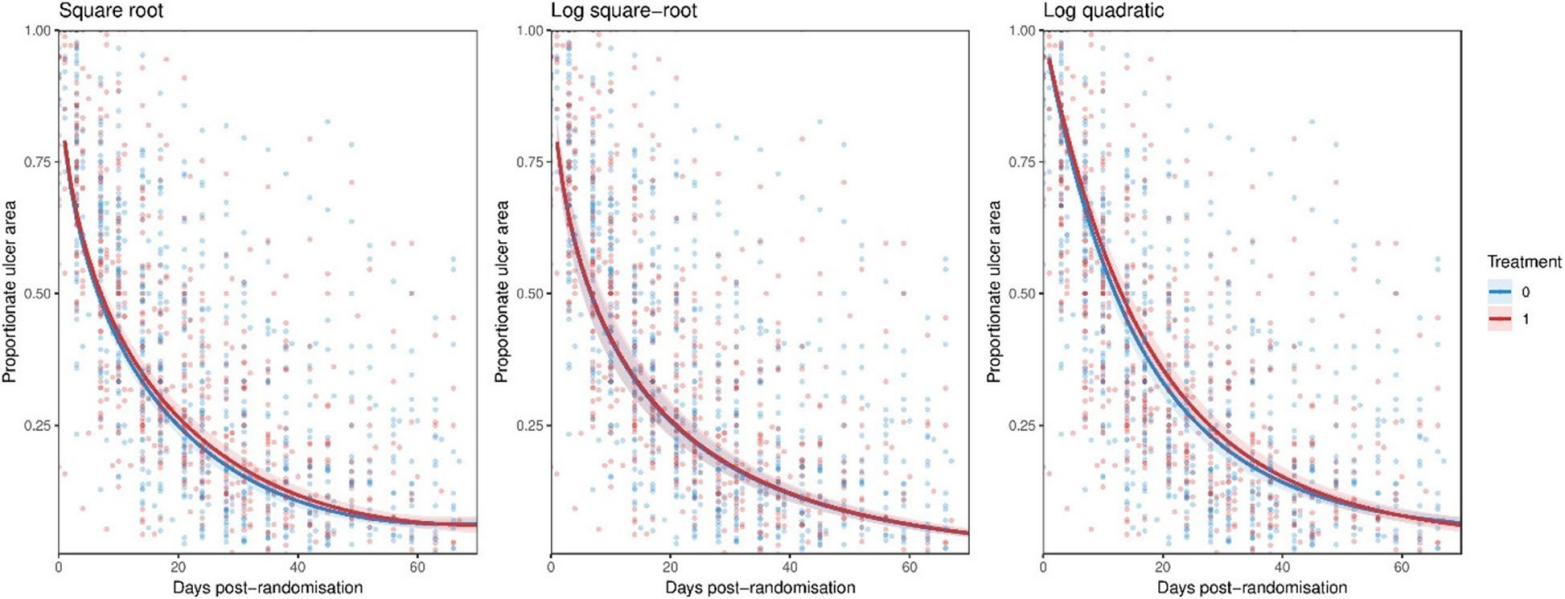
Estimated proportionate healing rate functions for the three best-fitting models using the ARANZ Automatic data. The lines show the estimated function for treatment and control groups with 95% credible intervals, the points are the data from the study

**Fig. 3:**
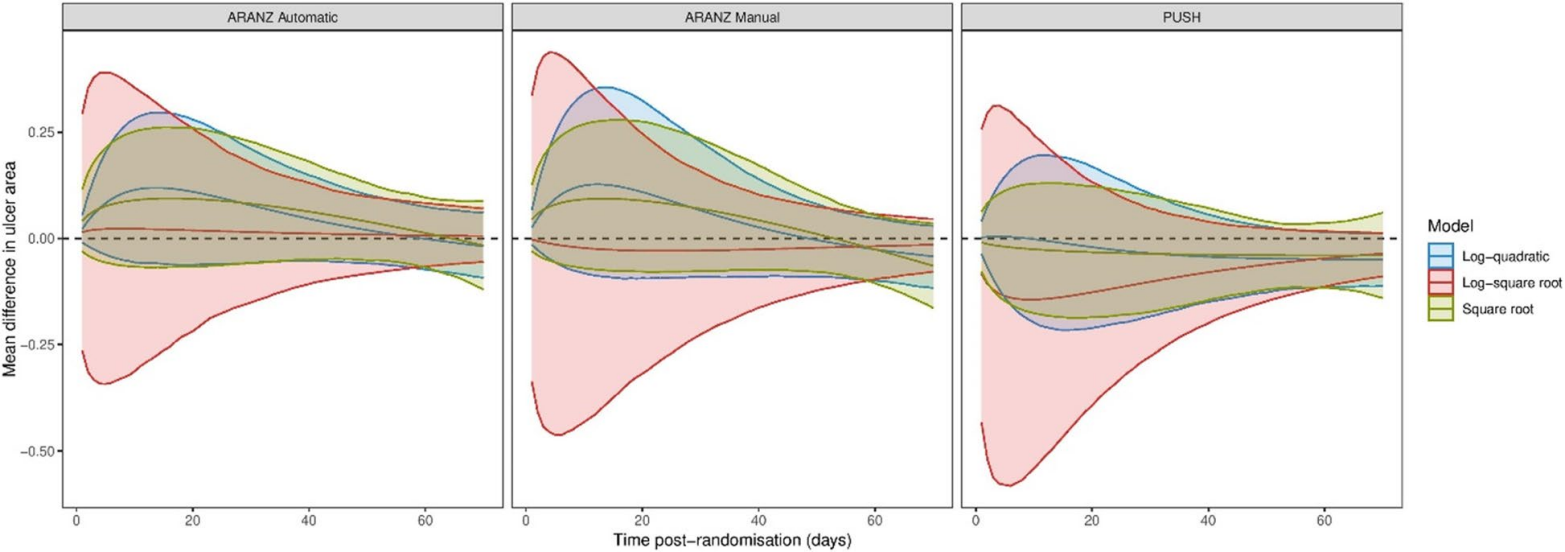
Estimated mean difference in ulcer area and 95% credible intervals derived from the three best-fitting models for all three sets of outcome data

We compared the estimated functions and treatment effects for the best three fitting models described above. Figure 2 shows estimated healing rate functions for treatment and control groups up to 70 days post-randomisation for ARANZ Automatic, overlayed on the data. Equivalent plots for the other outcomes are provided in the Supplementary Information. Notably, none of the functions start at one, and there is a large variance of ulcer sizes around the mean function. There is no clear difference between the estimated functions for the treatment and control groups. Figure 3 shows the estimated mean difference in ulcer area and 95% credible intervals for the three best-fitting models for all three sets of outcome data. The sign of the posterior mean difference (either favouring treatment or control) differs by both function and outcome type, however, all credible intervals overlap zero (and each other) for the whole course of the trial. The largest posterior mean difference was estimated using the log-square root model with the PUSH data nine days post-randomisation at -0.14 cm^2^ (95% credible interval: -0.55, 0.26). However, the same model estimated differences at the same time point of 0.02 cm^2^ (-0.32, 0.37) and − 0.02 cm^2^ (-0.44, 0.39) with the ARANZ Automatic and ARANZ Manual data, respectively, suggesting little evidence of effectiveness.

For comparison with non-parametric estimators, we examine estimates at 42 days post-randomisation, the primary end point of the original trial. The estimated mean differences in ulcer area using a non-parametric approach were − 0.01 cm^2^ (-0.77, 0.74), -0.44 cm^2^ (-1.64, 0.76), and 0.11 cm^2^ (-0.87, 1.08) for PUSH, ARANZ Automatic, and ARANZ Manual respectively. The equivalent figures for the square-root model were − 0.03 cm^2^ (-0.14, 0.06), 0.06 cm^2^ (-0.05, 0.17), and 0.03 cm^2^ (-0.07, 0.17).

## Discussion

There are multiple functions that may usefully describe the healing rate of a wound. However, there have been few studies to compare their performance using “real-world” wound healing data. Within a log-linear model, we identified that a square-root, log-square root, or log-quadratic healing rate function best fit data from a trial evaluating an intervention to promote ulcer healing. While the precise point estimates of the effect of the treatment differed between models and outcome data sets, all gave qualitatively similar conclusions about the (in-)effectiveness of the intervention.

In other settings and trials, it is possible that other functions than those we identified as best-fitting may better describe the data. We posit that large discrepancies in the relative healing rates would be unlikely between the data observed here and in other contexts. Indeed, the earliest paper we could identify on the topic of modelling wound healing, using data from frogs, also suggested the use of a square-root function. However, for different shaped wounds, different pathologies, or location on the body, the best fitting function may well differ. For example, the wounds in our data set may not well represent deeper, irregular, or “non-saucer shaped” wounds, like a pressure ulcer or a dehisced surgical wounds. We would advocate that a full model-based analysis includes a comparison of different candidate models, a good number of which we have described in this article. There are other non- and semi-parametric estimators of functions that may also be of interest, such as kernel-based estimators; there is a bias-variance trade-off involved with making stronger functional and parametric assumptions. However, our results indicated that the semi-parametric comparator did not perform as well as the functional alternatives.

We showed how the model estimates may be transformed into estimates of treatment effectiveness. The treatment effect varied by both time of assessment, type of model, and type of outcome measure. While no combination suggested strong evidence in favour of the intervention, the differences do suggest caution should be exercised when just considering a single time point and outcome measure in a trial evaluation. A model-based analysis would permit estimation of the effects across the course of the trial.

We argued that a log-linear model would be appropriate given assumptions about the measurement error inherent to measuring wounds. There are other plausible alternatives, such as an additive error on the linear scale. We conducted a brief exploratory analysis of alternative overall specifications, and found they performed significantly worse that the overarching model structure described here, and so were not investigated further. The trial that produced the data analysed in this article, [18] of which we were collaborators, included the mean difference in healing *rate* at 42 days post-randomisation as a primary treatment effect. This effect was estimated using a linear model of absolute ulcer area with a quadratic function of time post-randomisation. The trial analysis was planned before the analyses in this article were conducted, but given our results, we would now recommend against the model used by the trial. We would also recommend against comparing differences in healing *rate* as opposed to healing area for two reasons: differences in rates are hard to interpret clinically, mean differences in healing rate are complex to estimate given the need to average the first derivative with respect to time over the course of the trial. Despite these differences, at the time the trial report concluded there was little evidence of effectiveness of the intervention, but that there may be a small benefit. The new results we show here would suggest that even that conclusion may be too strong given how much it depends on specific assumptions about the healing process.

Model-based analyses can provide a principled way of combining large amounts of data from across the course of a trial. However, one must be careful not to allow the conclusions to depend heavily on modelling choices. A growing body of work discusses principled statistical “workflow” [19], a major component of which is model selection. For wound healing there are multiple plausible models: model comparison in terms of goodness of fit can be included as part of a trial protocol, which provides an opportunity to estimate a wide range of measures of effectiveness.

## Supplementary Information

Supplementary Material 1.

## Data Availability

Anonymised data are available from the authors on request.

## Abbreviation

RCT: Randomised controlled trial
