# Supplementary material for: Modelling wound area in studies of wound healing interventions
Source: BMC Med Res Methodol. 2024 Sep 16;24:206. doi: 10.1186/s12874-024-02326-y (PMC11403831; doi:10.1186/s12874-024-02326-y)
Supplement: Supplementary file 1 — Supplementary Material 1. [file 12874_2024_2326_MOESM1_ESM.docx]

**SUPPLEMENTARY INFORMATION**

**Other treatment effect estimators**

*Mean difference in healing rate per time* $\Delta t$ *at time* $T$

$$\delta_{3}^{'}\left( t \right)=E\left[ \frac{y_{i}\left( t+\Delta t \right)-y_{i}\left( t \right)}{\Delta t} | d=1 \right]-E\left[ \frac{y_{i}\left( t+\Delta t \right)-y_{i}\left( t \right)}{\Delta t} | d=0 \right]$$

$$=\frac{1}{\Delta t}E\left[ y_{i}^{*}\left( 0 \right) \right]\left( f_{1}\left( t+\Delta t \right)-f_{1}\left( t \right) \right)-\frac{1}{\Delta t}E\left[ y_{i}^{*}\left( 0 \right) \right]\left( f_{0}\left( t+\Delta t \right)-f_{0}\left( t \right) \right)$$

$$=\frac{1}{\Delta t}\exp\left( \beta_{0}+\frac{\omega^{2}+\tau^{2}}{2} \right)\left( f_{1}\left( t+\Delta t \right)-f_{1}\left( t \right)-f_{0}\left( t+\Delta t \right)+f_{0}\left( t \right) \right)$$

The function $f\left( t \right)$ is non-linear so the value of $\delta_{3}^{'}$ will differ by *time* $t$. To summarize the effect into a single estimate of treatment effect, we need to average the difference in healing rate over the course of the study:

$$\delta_{3}=\frac{1}{T}\int_{0}^{T} \delta_{3}^{'}\left( t \right)dt$$

For some of the parametric functions in Table 1, we can calculate this integral exactly. For example, for the square-root function we would have $\delta_{3}=\frac{T}{6}\left( 3\rho_{1}T+4\rho_{2}\sqrt{T}+6 \right)$. However, for other functions, including the log-linear, log-quadratic, and log-square root, there is no simple closed-form solution.

*Mean difference in proportionate healing rate per time* $\Delta t$ *at time* $T$

$$\delta_{4}=\frac{1}{\Delta t}\left( f_{1}\left( t+\Delta t \right)-f_{1}\left( t \right)-f_{0}\left( t+\Delta t \right)+f_{0}\left( t \right) \right)$$

*Survival Function*

The time to event outcomes can also be derived from the models described above. We make the assumption that a wound spontaneously heals whenever it is smaller than some size $\alpha$ since many of the models only allow for zero sized ulcers as time tends to infinity. The healing time of patient $i$ is then $T_{i}$. The survival function is $S\left( t_{0} \right)=\Pr\left( T_{i}>t_{0} \right)$ which we can rewrite as:

$$\Pr\left( y_{i}\left( t \right)>\alpha\right)=\Pr\left( y_{i}^{*}\left( 0 \right)f\left( t \right)u_{i}\left( t \right)>\alpha\right)=\Pr\left( y_{i}^{*}\left( 0 \right)u_{i}\left( t \right)>\frac{\alpha}{f\left( t \right)} \right)$$

Assuming $\log\left( y_{i}^{*}\left( 0 \right)u_{i}\left( t \right) \right)\sim N\left( \mu+\frac{\tau^{2}+\omega^{2}}{2},\tau^{2}+\omega^{2} \right)$ we can specify that:

$$S\left( t \right)=1-H\left( \frac{1}{f\left( t \right)};\beta_{0}-\log\left( \alpha\right),\tau^{2}+\omega^{2} \right)$$

Where $H\left( x;m,v \right)$ is the cumulative distribution function for a log-normal distribution with mean $m$ and variance $v$.

*Mean difference in the proportion of wounds healed by time T*

The mean difference in the proportion of wounds healed at a given time $T$ is just the difference in the value of the survival function

$$\delta_{5}=S_{1}\left( T \right)-S_{0}\left( T \right)=H\left( \frac{1}{f_{0}\left( t \right)};\beta_{0}-\log\left( \alpha\right),\tau^{2}+\omega^{2} \right)-H\left( \frac{1}{f_{1}\left( t \right)};\beta_{0}-\log\left( \alpha\right),\tau^{2}+\omega^{2} \right)$$
